# Rare case of real-time observation of paralytic deterioration after cervical dislocation in the hyperacute phase

**DOI:** 10.1186/s12891-022-05345-2

**Published:** 2022-05-02

**Authors:** Tsutomu Endo, Kota Suda, Takafumi Fukui, Satoko Matsumoto, Miki Komatsu, Masahiro Ota, Chikara Ushiku, Junichi Yamane, Akio Minami, Masahiko Takahata, Norimasa Iwasaki

**Affiliations:** 1 Hokkaido Spinal Cord Injury Center, Higashi-4, Minami-1, Bibai, Hokkaido 072-0015 Japan; 2 Department of Orthopaedic Surgery, Faculty of Medicine and Graduate School of Medicine, Hokkaido University, Kitaku Kita-15 Nishi-7, Sapporo, Hokkaido 060-8638 Japan

**Keywords:** Spinal cord injury, Dislocation, Quadriplegia, Complete paralysis

## Abstract

**Background:**

There have been no prior reports of real-time detailed records leading to complete quadriplegia immediately after fracture dislocation in high-energy trauma. Here, we report a case of cervical dislocation in which the deterioration to complete motor paralysis (modified Frankel B1) and complete recovery (Frankel E) could be monitored in real time after reduction in the hyperacute phase.

**Case presentation:**

A 65-year-old man was involved in a car accident and sustained a dislocation at the C5/6 level (Allen–Ferguson classification: distractive flexion injury stage IV). His paralysis gradually deteriorated from Frankel D to C 2 hours after the injury and from Frankl C to B 5 hours after the injury. His final neurological status immediately before reduction was Frankel B1 (complete motor paralysis with sensation only in the perianal region). Reduction was completed within 6 h and 5 min after injury, and spinal fusion was subsequently performed. The patient exhibited rapid motor recovery immediately after surgery, and was able to walk independently on postoperative day 14.

**Conclusions:**

This case suggests that there is a mixture of cases in which the spinal cord has not been catastrophically damaged, even if the patient has complete motor paralysis. Prompt reduction has the potential to improve neurological function in such cases.

## Background

Cervical dislocation fractures associated with injuries may escape irreversible spinal cord damage at the time of injury, and some cases of paralysis may recover with early dislocation reduction. However, even in the absence of catastrophic damage, paralysis can deteriorate from incomplete to complete within only a few hours of injury.

It is critical to make a prompt diagnosis and to implement neuroprotective interventions to protect neurological function, which is gradually lost in the hours immediately following spinal cord injury (SCI) [1]. In patients with cervical SCI, early decompression (within 24 h) has been reported to be associated with an American Spinal Injury Association (ASIA) disability scale grade two or more higher than late decompression (more than 24 h) [2]. However, the validity of early decompression and the eligibility of time limits remain uncertain.

Here, we report a rare case in which we were able to document in real time the deterioration to complete motor paralysis after cervical dislocation, and the rapid recovery of paralysis with reduction. We showed that even if the spinal cord is not catastrophically damaged at the time of injury, the remaining strangulation of the spinal cord can lead to complete paralysis. This suggests that among the cases of complete paralysis, there exists a mixture of cases in which paralysis can potentially improve. We believe that detailed time-course data reflecting neurological function is also useful information that contributes to the time limits of decompression.

## Case presentation

**Fig. 1:**
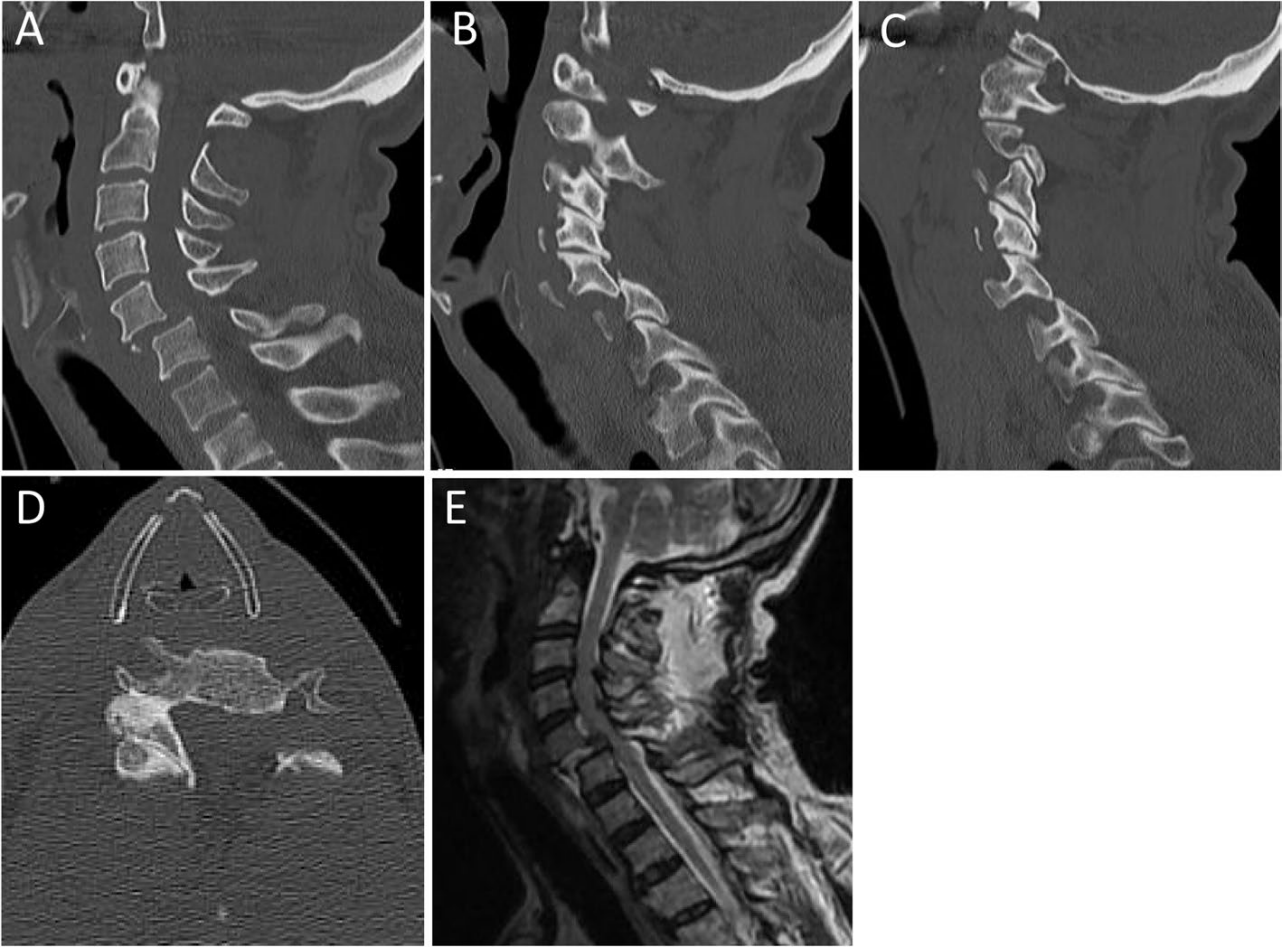
Preoperative CT scan showing a cervical dislocation at the C5/6 level: (**A**) central part of the sagittal reconstruction view, (**B**) right facet view, (**C**) left facet view, and (**D**) axial image. (**E**) Preoperative MRI showing severe spinal cord compression at the C5/6 level by the disc and the posterior supporting structure

A 65-year-old man was involved in a traffic accident while driving. Immediately after the injury, the patient was fitted with a cervical collar by paramedics, and his neck and trunk were firmly immobilized with a backboard. It took the paramedics approximately 3 hours to transport the patient directly to our hospital by ambulance, during which we were informed of the progress of paralysis by phone every hour. His paralysis status deteriorated from Frankel grade D to C 2 h after the injury and from Frankel grade C to B 5 h after the injury (Table 1). Upon arrival at our hospital, his neurological status was considered modified Frankel B1, indicating complete motor paralysis with sensation only in the perianal region. Following the CT and MRI scans, the patient was diagnosed with a dislocation at the C5/6 level (Allen–Ferguson classification: distractive flexion injury, stage 4) (Fig. 1).

**Table 1:** Modified Frankel grading system

**Grade A:** Complete: no motor or sensory function **Grade B:** Sensory only: some sensation preserved, no motor function **B1:** Touch sensation remains in only sacral lesion **B2:** Touch sensation remains in lower extremity **B3:** Pain sensation remains in sacral lesion or lower extremity **Grade C:** Motor useless: some sensory and motor function, but motor function not useful **C1:** Unable to flex the hip and knee from supine (Hip flexors 0–2) **C2:** Able to flex the hip and knee from supine (Hip flexors 3–5) **Grade D:** Motor useful: sensory function preserved, motor function but useful **D0:** MMTs of lower extremity are 4–5, but because of an acute phase, it is impossible to test the walking ability **D1:** Able to walk with a walker, but not practiced, usually use a wheelchair **D2:** Independent gait with a cane **D3:** Independent gait without a cane **Grade E:** Normal: normal sensory and motor function (hyperreflexia and numbness are permitted)

**Fig. 2:**
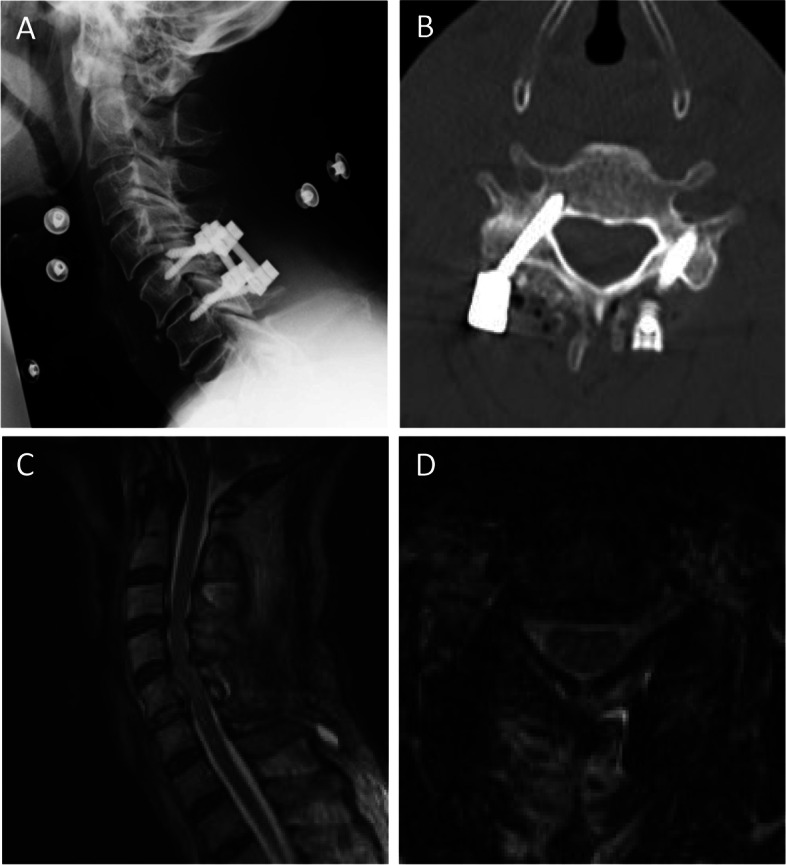
Postoperative X-ray (**A**), CT (**B**), MRI (**C** and **D**) shows successful reduction of dislocation, instrumentation, and decompression of the spinal cord

**Fig. 3:**
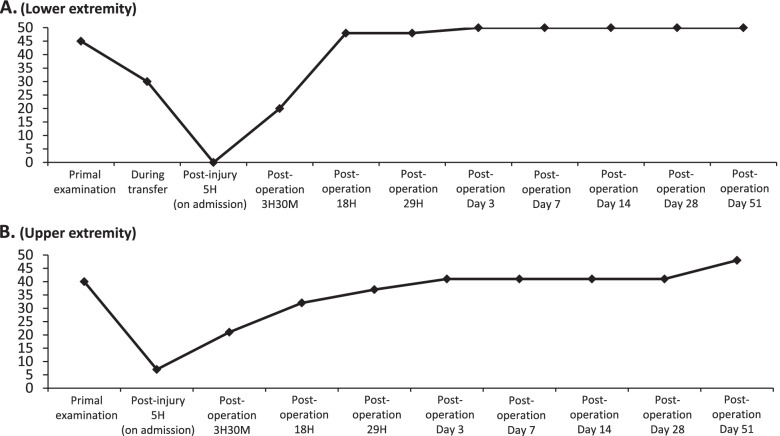
Graphs showing American Spinal Injury Association (ASIA) motor scores: the patients’ motor function in the lower (**A**) or upper (**B**) extremities deteriorated gradually after injury, and then recovered rapidly after surgery. The horizontal axis shows the time from injury to recovery. The vertical axis shows the sum of the strength of key muscles (0–5) of both lower extremities (L2, L3, L4, L5, and S1) and both upper extremities (C5, C6, C7, C8, and T1), respectively

After undergoing the minimum required evaluations, the patient was allowed to enter the operating room 54 min after arrival at our hospital. Surgery began 80 min after arrival (5 h and 54 min after injury), and reduction of the dislocation was performed approximately 11 min after the start of the surgery (6 h and 5 min after injury). C5/6 posterior fixation was performed with a pedicle screw and lateral mass screw system, and the surgery was completed in 55 min (Fig. 2). Soon after, the patient awoke from anesthesia, spontaneous limb activity was observed, and he rapidly recovered to grade 4 to 5 on manual muscle testing overnight. Three hours and 30 min postoperatively, his perception of touch and pain had completely recovered. His anal tone, bilateral anal wink, voluntary anal contraction, deep anal pressure, and bulbocavernosus reflex also improved. He had recovered sufficiently to walk independently 14 days after surgery and completely recovered to Frankel E 51 days after surgery (Fig. 3).

## Discussion

The aim of urgent reduction of cervical fracture dislocations is to protect the spinal cord and minimize secondary spinal cord injuries [2, 3]. There are two types of paralysis courses: complete paralysis caused by catastrophic spinal cord damage confirmed at the time of injury, and complete paralysis that occurs hours after injury despite being spared catastrophic damage from the primary injury. The latter is common in cervical spinal cord injuries caused by low-energy traumas.

Newton et al. [4] reported that when closed reduction was performed within 4 h after cervical dislocation sustained during a rugby game, 7 of 12 patients with complete motor paralysis (Frankel A or B) at the initial visit recovered to Frankel E (full recovery of lower limb muscle strength). Conversely, none of the patients fully recovered ambulatory abilities when closed reduction was performed > 9 h after injury. Rabinowitz et al. [5] demonstrated that neurological symptoms in beagle dogs improved within 6 h of experimental induction of spinal cord damage after nerve decompressive surgery. Considering these previous studies, the time limit for successful rescue of spinal cord function is estimated to be within 6 to 8 h after primary spinal cord injury.

Newton et al. [4] described not only the importance of the timing of dislocation reduction, but also differences in the degree of reversibility of spinal cord damage. Irreversible spinal cord damage is much more likely to be prevented for cervical fracture-dislocation associated with low-energy trauma incurred during an event such as a rugby game than for those associated with high-energy trauma such as traffic accidents. Newton et al. [4] suggested the following: 1) it is highly likely that fracture-dislocation caused by low-energy trauma is immune to irreversible changes in the spinal cord, and 2) if the dislocation is reduced within a short period of time, the possibility of avoiding secondary spinal cord damage and improving paralysis increases.

We speculated why the patient’s paralysis, which was classified as Frankel grade D immediately after the injury, rapidly deteriorated to Frankel B. From a microscopic perspective, one possible explanation could be the occurrence of cellular dysfunction or death during the acute phase of injury. These can be caused by ischemia, cell permeability, and pro-apoptosis due to disruption of the microvascular supply of the spinal cord within minutes after injury [6–8]. In addition, levels of pro-inflammatory cytokines, such as tumor necrosis factor (TNF) and IL-1β (interleukin 1 beta), which may exacerbate spinal cord injury, are increased in the spinal cord within minutes after injury [8, 9].

This case report had several limitations. First, we did not attempt closed reduction in this case. Thus, closed reduction and external fixation may have resulted in improvement of paralysis. However, it should be noted that closed reduction of cervical dislocation sometimes leads to worsening of paralysis [10, 11]. We performed open reduction and fixation because we considered surgery to be more reliable than closed reduction, and we were not certain whether closed reduction would provide sufficient recovery of lower-extremity muscle strength in patients more than 5 h after the injury. Second, it is unclear whether early treatment of spinal cord injuries, including high-energy trauma, will dramatically improve paralysis in all patients.

## Conclusions

Our experience in this case suggests that there is a mixture of cases in which the spinal cord has not been catastrophically damaged, even if the patient has complete motor paralysis. The fact that complete motor paralysis occurred 5 h after the injury, and that paralysis quickly recovered following reduction is also useful information in determining the time limit. Currently, there is no reliable way to distinguish which cases are salvageable and which are too late; as such, we believe that prompt reduction is desirable in all cases.

## Data Availability

Not applicable.
